# Integrated NV Center-Based Temperature Sensor for Internal Thermal Monitoring in Optical Waveguides

**DOI:** 10.3390/s25134123

**Published:** 2025-07-02

**Authors:** Yifan Zhao, Shihan Ding, Shuo Wang, Yiming Hu, Hongliang Liu, Zhen Shang, Yongjian Gu

**Affiliations:** 1 College of Physics and Optoelectronic Engineering, Ocean University of China, Qingdao 266100, China; zyf1140@stu.ouc.edu.cn (Y.Z.); wangshuo4634@stu.ouc.edu.cn (S.W.); hym7890@stu.ouc.edu.cn (Y.H.); yjgu@ouc.edu.cn (Y.G.); 2 Engineering Research Center of Advanced Marine Physical Instruments and Equipment of Ministry of Education, Ocean University of China, Qingdao 266100, China; 3 Qingdao Key Laboratory of Optics and Optoelectronics, Ocean University of China, Qingdao 266100, China; 4 Institute of Modern Optics, Nankai University, Tianjin 300350, China; 2120220310@mail.nankai.edu.cn (S.D.); drliuhl@nankai.edu.cn (H.L.); 5 China Ship Research and Development Academy, Beijing 100192, China

**Keywords:** optical waveguide, femtosecond laser direct writing, nitrogen-vacancy centers, temperature sensing

## Abstract

Color centers in solids, such as nitrogen-vacancy (NV) centers in diamonds, have gained significant attention in recent years due to their exceptional properties for quantum sensing. In this work, we demonstrate an NV center-based temperature sensor integrated into an optical waveguide to enable internal temperature sensing. A surface-cladding optical waveguide was fabricated in a diamond wafer containing NV centers using femtosecond laser direct writing. By analyzing the resonant peaks of optically detected magnetic resonance (ODMR) spectra, we established a precise correlation between temperature changes induced by the pump laser and shifts in the ODMR peak positions. This approach enabled temperature monitoring with a sensitivity of 1.1 $\mathrm{mK}/\sqrt{\mathrm{Hz}}$. These results highlight the significant potential of color centers in solids for non-contact, micro-scale temperature monitoring.

## 1. Introduction

Optical waveguides are structures designed to guide light along a specific path by confining it within a material with a higher refractive index than that of the surrounding material. An optical waveguide’s structure typically features small volumes, low-loss light transmission, a high photon density, and a compact geometry. Benefiting from these advantages, optical waveguides have been widely used in various applications, including optical communication [1,2], advanced sensing [3,4], nonlinear optics [5,6], compact light sources [7,8], and lasers [9,10]. As light-guiding structures, their compact size makes optical waveguides ideal for integration; however, this small size also means that when high photon densities are transmitted through the waveguide, the resulting temperature rise becomes significant [11]. Temperature increases caused by light can alter the refractive index, impacting the waveguide’s capacity to confine light beams effectively [12]. Prolonged exposure to high temperatures may also reduce the waveguide’s lifespan. In integrated optoelectronic devices, thermal effects can significantly reduce the efficiency of electron transfer. Additionally, local deformation caused by thermal effects plays a critical role, ultimately impacting the device’s overall performance and lifespan. For some more specific optical waveguide devices, such as the commonly used optical fiber, the maximum operating temperature is usually 85 degrees Celsius. The issue caused by high temperatures is usually progressive signal attenuation, and when the temperature exceeds the upper limit by 10 degrees Celsius, the aging speed will be doubled. Moreover, regarding array waveguide grating (AWG), its function is to channel multiple wavelengths of light into the same fiber, so the spectral drift caused by temperature changes significantly increases the insertion loss and crosstalk of the device. The initial step in managing thermal effects is to measure the thermal behavior of waveguides; however, research in this area remains limited. This gap is not due to a lack of importance of studying waveguide temperatures but rather stems from the inherent difficulty of directly measuring the internal temperature of waveguides. Conventional thermometers often fail in this context due to an insufficient spatial resolution.

Recently, the use of confocal luminescence thermometry has been reported for measuring temperatures in waveguides [13,14], effectively addressing challenges such as the spatial resolution, sub-degree thermal sensitivity, and non-contact measurement. Building on this advancement, we propose a new approach that leverages the fluorescence of NV centers in diamonds to directly measure the temperature within the waveguide. NV centers in diamonds are among the most widely utilized solid-state quantum systems [15]. Their electron spin states can be manipulated using ODMR, making them highly promising for applications in quantum technology. Moreover, the electron Hamiltonian of NV centers is sensitive to various external fields, enabling diverse and well-established applications in quantum sensing, including the sensing of magnetic fields [16,17], temperatures [18,19], orientations [20,21], and more.

In this work, we used NV centers as temperature sensors to directly measure the temperature within the surface-cladding optical waveguide in a diamond wafer. The waveguide was fabricated by femtosecond laser direct writing. It is important to clarify that the NV centers inside the waveguide were not generated by the femtosecond laser [22]. The femtosecond laser was employed to fabricate the waveguide in the diamond, and its action was confined to the boundaries of the waveguide [23,24]. A 532 nm laser served as both the pump laser within the waveguide and the excitation laser for the NV centers. By analyzing the resonant peaks of the ODMR spectra of the NV centers within the waveguides, we successfully implemented temperature monitoring with a sensitivity of 1.1 $\mathrm{mK}/\sqrt{\mathrm{Hz}}$. Although this experiment was conducted using NV centers in diamonds, many other color centers in solids exhibit similar properties. This suggests that this approach could be widely applied to various types of optical waveguides made from different materials.

## 2. Experiments

**Figure 1:**
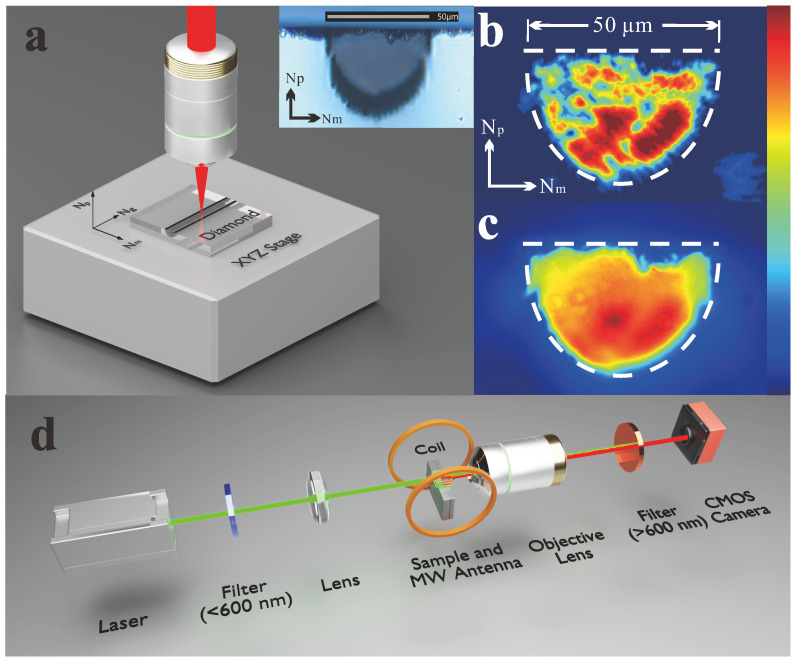
Schematic diagram and characteristics of the waveguide. (**a**) Schematic diagram of femtosecond laser direct writing and a cross-sectional microscope image of the waveguide. (**b**,**c**) The near-field model profiles of the waveguide at laser (532 nm) and fluorescence (600–800 nm) wavelengths, respectively. The dashed line marks the spatial boundaries of the structure. (**d**) Schematic diagram of the end-face coupling arrangement for waveguide characterization and ODMR.

A polished diamond wafer with a size of 3 × 3 × 0.3 mm^3^ was used in this research, which was purchased from Asia Carbon (Shenzhen) Technology Co., Ltd., Shenzhen, China. It was a type IIa diamond synthesized using the CVD method. The diamond product model was Q-DNV-S, with a nitrogen concentration of about 0.5 ppm and NV center concentration of around $6\times 10^{-3}$ ppb. The optical waveguide in the diamond wafer was fabricated using femtosecond laser direct writing technology. It was a half-circle surface-cladding optical waveguide with a width of 50 μm, extended 3 mm in length, immediately beneath the surface as shown in Figure 1a. The waveguide loss at 532 nm was 8.44 dB, including the propagation and coupling losses. The femtosecond laser employed for direct writing was generated by an amplified Ti laser system (Astrella, Coherent Inc., Santa Clara, CA, USA) and operated at a 1 kHz repetition rate, with a pulse width of 100 fs, a pulse energy of 5 mJ, and a central wavelength of 800 nm. A long-working-distance objective lens (50×, NA = 0.55) focused the laser beam on the top surface of the diamond wafer, with a single pulse energy of 33 nJ achieved using a Watt Pilot motorized attenuator. The waveguide fabrication process is shown in Figure 1a and used a scanning speed of 0.1 m/s. Figure 1b presents a cross-sectional microscope image of the surface-cladding waveguide.

An end-face coupling system, as shown in Figure 1d, was employed for both the collection of both excitation and fluorescence from the NV centers in the waveguide. A continuous 532 nm laser with a maximum output power of 1 W was used to excite the NV centers to a higher energy state and to heat the waveguide to elevate the temperature. The laser passed through a low-pass filter with a cutoff wavelength of 600 nm before exiting the waveguide, effectively eliminating longer-wavelength light. Input coupling was achieved using a biconvex lens (f = 25 mm), while output coupling was performed with a 20× objective lens (NA = 0.4) to collect the fluorescence. A high-pass filter with a cutoff wavelength of 600 nm was used to block any residual laser light. The modal profile of the waveguide was captured using a CMOS camera (INOVANCE IC0600GM, with a spectral range of 400–1000 nm), with the near-field model profile shown in Figure 1b,c. The waveguide was multimodal, functioning at both the laser wavelength (532 nm) and the fluorescence wavelength (600–800 nm), and the boundaries of the near-field images were in good agreement with the geometries of the structure. It can be observed that the NV centers in the waveguide were uniformly excited, the laser was effectively confined within the waveguide, and the fluorescence used for subsequent sensing was exclusively from within the waveguide. A spectrometer (an Andor SR-500i-A with a CCD DV420A-OE) was used to measure the PL spectra of the NV centers. A confocal Raman system (a Thermo Scientific DXR3xi using a continuous 532 nm laser as the probe light) was used to obtain the Raman spectra mapping results.

An ODMR experiment was employed to measure the internal temperature of the waveguide. The principle of this experiment was based on the temperature dependence of the zero-field splitting (ZFS) of NV centers. The shift in the resonance frequency with the temperature is denoted as dD/dT, with a typical value of 74 kHz/K [25]. However, this value may vary slightly between different NV centers. Therefore, the calibration of dD/dT was required prior to temperature measurements. For the ODMR measurement, the sample was placed on a self-made microwave antenna, which met the requirements of ODMR. The microwave source (Rohde & Schwarz, SMB100B) provided microwave signals in the range of 0–3 GHz with a maximum power of 30 dBm, and the external microwave amplifier (Minicircuits, ZHL-16W-43-S+) had a gain of 45 dB. A bias magnetic field was supplied by a pair of Helmholtz coils powered by a constant current supply, providing a magnetic field with a magnetic induction between 0 and 15 Gs.

## 3. Results and Discussion

**Figure 2:**
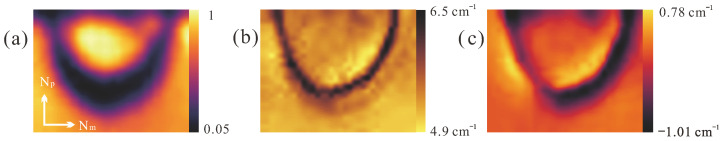
Raman mapping of the polished end-facet of an fs-DLW diamond waveguide geometry, showing the 1332 ${\mathrm{cm}}^{-1}$ Raman peak: (**a**) the peak Raman intensity, (**b**) peak width, and (**c**) peak frequency shift.

To investigate the effect of laser-induced tracks in the guiding region, the Raman properties across the waveguide cross-section were analyzed. Figure 2a–c are the two-dimensional scanning images of the Raman intensity, peak full width at half maximum (FWHM), and peak frequency shift at a peak of 1332 ${\mathrm{cm}}^{-1}$, respectively. The Raman spectra revealed a decrease in the intensity, the broadening of the FWHM, and a shift in the peak position within the laser-irradiated region. These changes indicate the presence of structural damage and crystalline lattice expansion in the track region, leading to a reduction in the refractive index along the depressed cladding structure [26]. In contrast, the guiding core and the surrounding substrate exhibited nearly unchanged Raman features, suggesting that the laser writing had a minimal impact on the crystal in these areas. As a result, light could be well confined within the entire compact waveguide structure.

**Figure 3:**
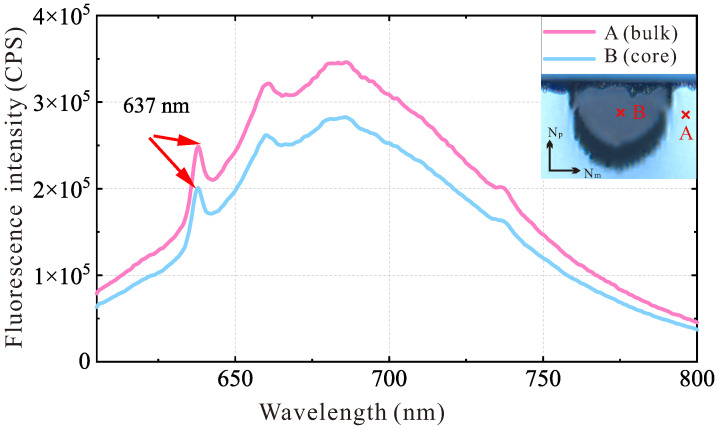
Fluorescence spectra at point A (bulk) and point B (core).

Since the diamond sample used in this work did not undergo electron irradiation or annealing treatment after the growth process, a photoluminescence (PL) measurement was performed to assess the presence of NV centers in the diamond. The energy gap between the ground and excited states of the NV centers was approximately 1.95 eV. This corresponded to the zero-phonon line (ZPL) at 637 nm, observed in the PL spectrum shown in Figure 3. The PL measurement confirmed the presence of NV centers in the diamond. Meanwhile, spectra measured within the waveguide (core) and outside of the waveguide (bulk) indicated that the properties of the guiding region had not been modified obviously during the laser writing. The peak observed around 735 nm corresponded to intrinsic silicon vacancy centers in the diamond [27]. These intrinsic defects had no effect on the subsequent ODMR.

**Figure 4:**
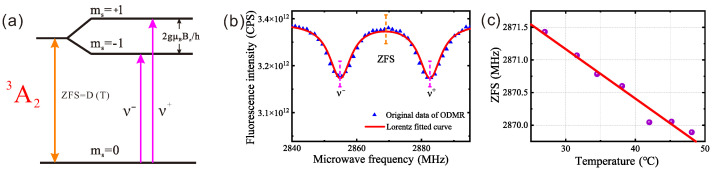
(**a**) The ground-state spin levels of the NV centers; (**b**) the ODMR spectrum under a bias magnetic field measured at 45 °C; (**c**) the temperature reliability of the thermometer: *D* as a function of the temperature. (Celsius is used as the temperature unit to visually display the temperature).

The ground-state spin levels of the NV centers are shown in Figure 4a. The energy gaps between the $|\pm 1\rangle$ and $|0\rangle$ states were defined as *D*, or the zero-field splitting (ZFS), which was 2.78 GHz in the absence of a magnetic field. In a magnetic field, the $|\pm 1\rangle$ states split into $|+1\rangle$ and $|-1\rangle$, with the corresponding energy gaps splitting into $\nu^{-}$ and $\nu^{+}$. In this case, D was equal to the average of $\nu^{-}$ and $\nu^{+}$. When a continuous microwave signal was applied at the ZFS frequency, electrons in a $|0\rangle$ spin state were driven to transition into $|\pm 1\rangle$ states, causing a decrease in the PL intensity, referred to as ΔPL. By recording the ΔPL/PL ratio or PL intensity as a function of the microwave frequency, optically detected magnetic resonance was performed [28]. Numerous studies have demonstrated that *D* is temperature-dependent [25], enabling NV centers to be used for temperature sensing. At room temperature, the relationship between *D* and the temperature *T* is approximately linear, as reported in the literature. The typical value for dD/dT is 74 kHz/K. However, it can fluctuate between 60 and 80 kHz/K due to variations in individual diamond samples. Therefore, measuring dD/dT is essential for accurate temperature sensing. To reduce the broadening of resonance peaks caused by spin-orbit coupling and crystal field effects within the diamond lattice [29], ODMR measurements were conducted in a weak magnetic field of 1 mT. The ODMR spectrum measured at 45 °C in a weak bias magnetic field is shown in Figure 4b. *D* was obtained by averaging the $\nu^{-}$ and $\nu^{+}$ transitions. By measuring *D* at different temperatures, the relationship between *D* and *T* was determined, as shown in Figure 4c. A controlled heating sheet was employed to achieve the required temperatures during the measurements. From the linear fit of the data in Figure 4c, the temperature coefficient, dD/dT, was calculated to be 75.74 kHz/K in this experiment.

**Figure 5:**
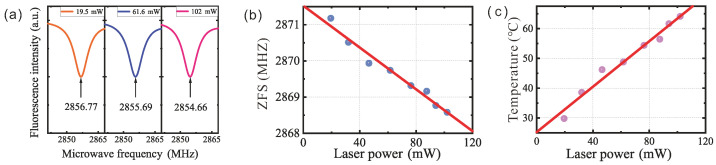
Temperature measurement in waveguide: (**a**) resonance frequency ($\nu^{-}$) at different laser powers, (**b**) ZFS value, *D*, as function of laser power, and (**c**) temperature as function of laser power.

Figure 5a shows the ODMR peak $\nu^{-}$ at different input laser powers. As the laser power increased, the ODMR peaks shifted to the left, indicating that *D* decreased. This confirms that a higher laser power induces internal heating in the waveguide. Figure 5b illustrates the function between the laser power and *D*. Since *D* had a linear relationship with *T*, the data were transformed to show the relationship between the laser power and *T*, as shown in Figure 5c. The highest temperature during the experiment, around 60 °C, was observed with the maximum laser power, about 100 mW, and the fitted ratio was 0.38 K/mW. This result confirms that the pump laser caused the temperature increase, which was measured by the NV center temperature sensor.

Here, we discuss the sensitivity of the NV center temperature sensors. For the CW-ODMR used in this experiment, its sensitivity was determined using the following formula [30]:

(1) $$\eta_{T}=\frac{4}{3\sqrt{3}}\cdot \frac{1}{dD/dT}\frac{\Delta \nu }{C\sqrt{R}},$$

where Δν is the FWHM of the ODMR peaks, *C* is the ODMR contrast, and *R* is the PL photon count rate. Δν and *C* could be read directly from the ODMR spectrum and were around 10 MHz and 4.5%, respectively, in this experiment. The photon counting rate clearly increased with an increase in the laser power. The highest luminescence power in this experiment corresponded to 1.22 μW. The sensitivity based on the maximum fluorescence power was calculated to be around 1.1 $\mathrm{mK}/\sqrt{\mathrm{Hz}}$.

## 4. Conclusions

In this work, we presented a method for temperature monitoring within optical waveguides embedded inside solid materials, utilizing color centers. By employing femtosecond laser direct writing, we fabricated a waveguide structure in a commercially available diamond wafer. Through ODMR measurements, we established a clear relationship between the shifts in the ODMR peak positions and the temperature changes induced by the pump laser. The sensitivity of the NV center-based sensor was determined to be 1.1 $\mathrm{mK}/\sqrt{\mathrm{Hz}}$, enabling accurate temperature measurements within the waveguide. We observed that a higher laser power increased the temperature within the waveguide, confirming the role of the pump laser in inducing local heating. The temperature coefficient, dD/dT, was found to be 75.74 kHz/K, further solidifying the potential of NV centers for use in precise temperature sensing. These findings highlight the NV center-based sensor’s ability to monitor the temperature in integrated photonic systems with a high spatial resolution and sensitivity, offering a powerful tool for thermal management in future optoelectronic and quantum devices. Furthermore, the approach used here can be extended to other color centers in solids, broadening its applicability in various material platforms for micro-scale, non-contact temperature sensing.

## Data Availability

The data will be available on request.

## Abbreviations

The following abbreviations are used in this manuscript:

NV	Nitrogen-vacancy
FsLDW	Femtosecond laser direct writing
ODMR	Optically detected magnetic resonance
